# 608. Risk Factors of Pseudomonal Wound Infections in Burn Patients

**DOI:** 10.1093/jbcr/irag033.419

**Published:** 2026-04-06

**Authors:** Shannon Kuhrau, Sylvia Karpio, Kaley Deichstetter

**Affiliations:** Loyola University Medical Center, Maywood, IL; Loyola University Medical Center, Maywood, IL; Loyola University Medical Center, Maywood, IL

## Abstract

**Introduction:**

Burn wounds infections with *P. aeruginosa* are common and are associated with higher mortality rates and risk of invasive infection. With the rise of multi-drug resistant organisms and their associated increased morbidity and mortality in burn patients – the need of appropriately targeted antibiotics for empiric treatment is vital to limit this risk. The purpose of this study was to identify risk factors associated with pseudomonal infections to best identify targeted use of broad spectrum antibiotics.

**Methods:**

Retrospective chart review of patients with culture positive wound infections admitted to the burn unit from January 2019 through October 2024. Patients with culture-positive wound infections positive for *P. aeruginosa* were compared to non-pseudomonal culture positive wound infections. Culture positivity included both tissue cultures and blood cultures with a presumed wound infection source. Baseline characteristics were collected and included total body surface area (TBSA) burned, mechanism of burn, presence of inhalation injury, type of grafting, and time to infection. A multivariable logistic regression was conducted to determine independent risk factors of pseudomonal wound infections.

**Results:**

A total of 93 patients were included in the study: 47 with *P. aeruginosa*-positive infections versus 46 with other organisms. There were significant differences in the baseline characteristics between the pseudomonal vs. non-pseudomonal group in the univariate analysis, this included: TBSA (30% vs. 10.5%; *p*<.01), BMI (29.2 vs 26.4 kg/m2; *p*=.02), ICU status (91.5% vs. 67.4%), total number of operating room visits (5 vs 2; *p*<.01), and time to culture positivity (398.5 vs. 182.3 hours; *p*<.01). In the multivariable logistic regression, both BMI and time to culture positivity were determined to be independent risk factors.

**Conclusions:**

Time to culture positivity and BMI appear to be independent risk factors for the development of pseudomonal wound infections in burn patients. Empiric anti-pseudomonal antibiotics should be considered at a minimum in these patients. Results are limited by no universal burn wound infection diagnostic criteria and our study’s necessity of positive tissue culture results.

**Applicability of Research to Practice:**

Identifying which patients would benefit from targeted pseudomonal coverage is important to limit unwarranted broad-spectrum antibiotics and minimize risk of multidrug resistance.

**Funding for the study:**

N/A.

